# A Systematic Evaluation of the Two-Component Systems Network Reveals That ArlRS Is a Key Regulator of Catheter Colonization by *Staphylococcus aureus*

**DOI:** 10.3389/fmicb.2018.00342

**Published:** 2018-03-07

**Authors:** Saioa Burgui, Carmen Gil, Cristina Solano, Iñigo Lasa, Jaione Valle

**Affiliations:** Laboratory of Microbial Pathogenesis, Navarrabiomed-Universidad Pública de Navarra (UPNA)-Complejo Hospitalario de Navarra (CHN), Instituto de Investigación Sanitaria de Navarra, Pamplona, Spain

**Keywords:** two-component systems, *Staphylococcus aureus*, implants, biofilm, PNAG, *arlRS*

## Abstract

Two-component systems (TCS) are modular signal transduction pathways that allow cells to adapt to prevailing environmental conditions by modifying cellular physiology. *Staphylococcus aureus* has 16 TCSs to adapt to the diverse microenvironments encountered during its life cycle, including host tissues and implanted medical devices. *S. aureus* is particularly prone to cause infections associated to medical devices, whose surfaces coated by serum proteins constitute a particular environment. Identification of the TCSs involved in the adaptation of *S. aureus* to colonize and survive on the surface of implanted devices remains largely unexplored. Here, using an *in vivo* catheter infection model and a collection of mutants in each non-essential TCS of *S. aureus*, we investigated the requirement of each TCS for colonizing the implanted catheter. Among the 15 mutants in non-essential TCSs, the *arl* mutant exhibited the strongest deficiency in the capacity to colonize implanted catheters. Moreover, the *arl* mutant was the only one presenting a major deficit in PNAG production, the main exopolysaccharide of the *S. aureus* biofilm matrix whose synthesis is mediated by the *icaADBC* locus. Regulation of PNAG synthesis by ArlRS occurred through repression of IcaR, a transcriptional repressor of *icaADBC* operon expression. Deficiency in catheter colonization was restored when the *arl* mutant was complemented with the *icaADBC* operon. MgrA, a global transcriptional regulator downstream ArlRS that accounts for a large part of the *arlRS* regulon, was unable to restore PNAG expression and catheter colonization deficiency of the *arlRS* mutant. These findings indicate that ArlRS is the key TCS to biofilm formation on the surface of implanted catheters and that activation of PNAG exopolysaccharide production is, among the many traits controlled by the ArlRS system, a major contributor to catheter colonization.

## Introduction

The extensive use of indwelling devices such as prosthetic joints, heart valves, and intravascular catheters in hospitalized patients has increased the incidence of device related infections (DRI) (Hogan et al., 2015). DRI constitute a growing healthcare concern because they are associated with a high morbidity and mortality and also with increased costs in health care systems. *Staphylococcus aureus* is a frequent etiological agent of DRI, especially those related with intravascular catheters, prosthetic joints, vascular grafts and pacemakers (Kluytmans et al., 1997; Lowy, 1998; Ellis et al., 2014).

The success of *S. aureus* as a pathogen depends on the combined action of multiple factors. On one hand, *S. aureus* produces a wide array of cell surface and secreted virulence factors such as exoenzymes (coagulase, lipases, and proteases), toxins (cytolytic toxins, enterotoxins, exfoliative toxins) and immune evasion mechanisms (protein A) (Foster et al., 2014). Cell surface virulence factors allow the organism to colonize the host through adhesion to mucosal surfaces and to interfere with normal immune system functions. Secreted factors, including exoenzymes and exotoxins, allow the organism to spread into surrounding tissues and access nutrients through cell damage. On the other hand, *S. aureus* exhibits a great capacity to adapt to many different environments including almost every organ of the human body (lung, heart, blood, bone, skin, muscles, eye, joints, and intestinal tract). For that, *S. aureus* possesses efficient signal transduction systems that facilitate the integration of environmental stimuli and adjust the cellular physiology in response (Novick, 2003; Cheung et al., 2004; Haag and Bagnoli, 2016). The sensory machinery of *S. aureus* is composed of the two-component systems (TCS) network, a Ser/Thr protein kinase, two Ser/Thr phosphatases and a c-di-AMP cyclase (Dac) and a phosphodiesterase (GpdP) involved in c-di-AMP metabolism. As regards the TCS network, most *S. aureus* strains contain 16 TCSs, with one of them being essential for bacterial viability (Kuroda et al., 2001). In a canonical TCS, extracellular stimuli induce the autophosphorylation of a histidine kinase (HK). The activated HK transfers the phosphoryl group to a conserved aspartate residue present on the response regulator (RR). The phosphorylation of the RR activates an output domain, which can then effect changes in cellular physiology often by regulating gene expression (Stock et al., 2000).

When *S. aureus* reaches the surface of an implanted medical device, attachment to the surface occurs through interactions between bacterial adhesins and plasma proteins deposited on the implant’s surface. Once attached, *S. aureus* needs to adjust bacterial physiology to the sessile lifestyle. For that, *S. aureus* produces an extracellular matrix mainly composed of exopolysaccharides, proteins and extracellular DNA (O’Gara, 2007; Rice et al., 2007). The exopolysaccharide PNAG, also named PIA (polysaccharide intercellular adhesin) is a major component of the staphylococcal biofilm matrix. PNAG, β-1,6-linked *N*-acetylglucosamine, is synthetized by the enzymes encoded in the *icaADBC* operon whose expression is tightly regulated by the transcriptional repressor IcaR. Some strains of *S. aureus* make use of extracellular proteins to build the biofilm matrix by interacting with eDNA and polysaccharides or in some cases, through polysaccharide-independent mechanisms (Cucarella et al., 2001; Corrigan et al., 2007; O’Neill et al., 2008; Vergara-Irigaray et al., 2009; Taglialegna et al., 2016). Although the molecular determinants underlying the choice of either a polysaccharide or protein-based biofilm matrix are not well understood, it is assumed that environmental signals determine the composition of the biofilm matrix (Vergara-Irigaray et al., 2009). Attempts to identify the specific TCSs involved in the biofilm formation process of *S. aureus in vitro* have been previously performed (Toledo-Arana et al., 2005). However, identification of TCSs important for colonization of implanted medical devices *in vivo* remains to be elucidated.

In this study, we used a collection of single mutants in each non-essential TCS of *S. aureus* and a murine subcutaneous catheter infection model to identify the TCSs controlling the production and composition of the biofilm matrix on the surface of the implanted device. Our results revealed that *arlRS*, and in a lower extent *agr* and *srrAB* mutants, have a significant decreased capacity in colonize the surface of implanted catheters. In the *arl* mutant background, expression of the *icaADBC* operon restores the capacity to colonize the catheter, indicating that the PNAG exopolysaccharide is required for efficient colonization of implanted catheters. This result is central to the development of novel therapeutic approaches to specifically target biofilm related infections.

## Materials and Methods

### Oligonucleotides, Plasmids, Bacterial Strains, and Culture Conditions

Bacterial strains, plasmids and oligonucleotides (Stabvida) used in this study are listed in Supplementary Tables S1, S2. *Escherichia coli* strains were grown in LB broth (Conda-Pronadisa). *S. aureus* strains were grown in trypticase soy broth (TSB) (Conda-Pronadisa), TSB supplemented with glucose (TSBg) in strain ISP479r or NaCl 3% (TSB-NaCl) in the case of strain 132. When required for selection, medium was supplemented with appropriate antibiotics at the following concentrations: erythromycin (Em), 1.5 and 10 μg ml^-1^; ampicillin (Amp), 100 μg ml^-1^.

### DNA Manipulations and Bacterial Transformation

General DNA manipulations were performed using standard procedures. Plasmids were purified using the NucleoSpin Plasmid miniprep kit (Macherey-Nagel) according to the manufacturer’s protocol. FastDigest restriction enzymes and Rapid DNA ligation kit (Thermo Scientific) were used according to the manufacturer’s instructions. Plasmids were transformed into *E. coli XL1-Blue* strain (Stratagene) and *S. aureus* by electroporation, using previously described protocols (Cucarella et al., 2001). Staphylococcal electrocompetent cells were generated as previously described (Schenk and Laddaga, 1992).

### Allelic Exchange of Chromosomal Genes

We used a collection of single mutants in each TCS constructed in *S. aureus* MW2 (listed in Supplementary Table S1) by Villanueva et al. (2018) as follows. Fragments of at least 500 bp that flanked the left (primers A and B) and right sequences (primers C and D) of the region targeted for deletion were amplified by PCR. The PCR products AB and CD were used to obtain an overlapping PCR product named AD, which was cloned into the shuttle vector pMAD (Arnaud et al., 2004) or pMAD_lic. A LIC-modified pMAD vector was constructed in order to enable efficient directional cloning without restriction enzyme digestion or ligation reactions (Alanidis and Dejong, 1990). To create the pMAD_lic vector we oligomerized the primers pMAD_lic (EcoRI) and pMAD_lic (BamHI) listed in Supplementary Table S2, and cloned the double DNA strand dimer into pMAD using EcoRI and BamHI restriction enzymes. To produce specific non-complementary overhangs in the pMAD_lic vector, the ApaI linearized plasmid was treated with T4 DNA Polymerase (Novagen) in the presence of dTTP (Novagen) for 30 min at 22°C. PCR products with complementary overhangs were created using Phusion enzyme (Thermo Scientific) by building appropriate 5′ extensions into the primers. The PCR products were purified and then treated with T4 DNA Polymerase in the presence of dATP (Novagen) at 22°C. After 30 min the enzyme was inactivated. To anneal the insert into the pMAD_lic vector, the mix of vector and insert was incubated for 5 min at 22°C and then, EDTA (6.25 mM) was added and a further incubation of 5 min at 22°C was applied. To express the *ica* operon and *mgrA* gene under de cadmium promoter, we amplified the cadmium inducible promoter from pCN51 plasmid (*P*_cd_) (Charpentier et al., 2004) and two fragments of at least 500 bp that flanked the left (primers A and B) and right sequences (primers C and D) of the start of the mRNA of both genes. Three fragments were fused by overlapped PCR. The purified fragment was cloned into the pMAD_lic plasmid. Allelic exchange in the absence of a selection marker was performed as previously described (Valle et al., 2003). White colonies, which no longer contained the pMAD plasmid, were tested to confirm the replacement by PCR using primers E and F (Supplementary Table S2). Note that cadmium was not used for the expression of *P*_cd_ since the leakage of expression of the *P*_cd_ promoter was sufficient to express PNAG and MgrA.

### PNAG Detection

Overnight cultures of the strains tested were diluted 1:40 in the appropriate medium, and 2 ml of this cell suspension was used to inoculate sterile 24-well polystyrene microtiter plates (Sarstedt). After 16 h cell-surface PNAG production was detected as described previously (Valle et al., 2003). 5 μl of the extract or 5 μl of a dilution of the purified extract were spotted on a nitrocellulose filter using a Bio-Dot microfiltration apparatus (Bio-Rad), blocked overnight with 5% skim milk in phosphate-buffered saline with 0.1% Tween 20, and incubated for 2 h with an anti-PNAG antibody diluted 1:10,000 (Maira-Litrán et al., 2002). Bound antibodies were detected with peroxidase-conjugated goat anti-rabbit immunoglobulin G anti-bodies (Invitrogen) 1:5,000.

### Generation of *ica* and *mgrA* Transcriptional Fusions With Gfp

To obtain an *ica* and *mgrA* transcriptional fusions, we amplified the *ica* promoter using primers AU59 and AU76 and the *mgrA* promoter using primers PmgrA-fw and Pmgra-rv (Supplementary Table S2) and cloned in pCN52 plasmid (Charpentier et al., 2004), giving pCN52-P_*ica*(*BS*)-_gfp and pCN52-P_mgrA_-gfp plasmids, respectively. Plasmids were transformed in *S. aureus* wild type strains and *arl* mutants. To analyze *ica* and *mgrA* expression, total protein extracts were recovered and analyzed by SDS-PAGE and Western blot Gfp was detected using anti-GFP [Living Color^®^ A.v. Monoclonal Antibody (JL-8), Clontech] antibodies diluted 1:2,500 in 0.1% PBS-Tween 5% skim-milk. Peroxidase-conjugated goat anti-mouse Immunoglobulin G (Invitrogen) diluted 1:5000 in 0.1% PBS-Tween 5% skim-milk were used as secondary antibodies.

### RNA Extraction and Northern-Blot

*Staphylococcus aureus* 132 and *arl* strains were grown in 10 ml of TSB-NaCl at 37°C under static conditions for 5 h. RNA extraction was performed as described previously (Lasa et al., 2011). Northern blots were performed as described (Ruiz de Los Mozos et al., 2013). Briefly, 8 μg of total RNA was separated in precast agarose gels (Sigma). RNAs were blotted onto Nytran membranes (0.2-μm pore size) (Amersham Biosciences), UV cross-linked, prehybridized in ULTRAhyb solution (Ambion) at 65°C, and labeled with strand-specific riboprobes specific for *icaA, icaC*, and *icaR* (Supplementary Table S2). Membranes were washed and autoradiography images were registered at different exposition times according to each gene.

### Protein Tagging and Immunodetection Analysis

Transfer of the 3xFlag sequence into IcaC was achieved by recombination using plasmid pMADicaC-3xFlag. To construct pMADicaC-3xFlag the C-terminal region of *icaC* was amplified using primers CFlag1 and CFlag2 (Supplementary Table S2). The CFlag2 primer contains 66 nt that code for the 3xFlag sequence. Strains containing IcaC-3xFlag were grown in TSB-NaCl or TSBg at 37°C under static conditions for 5 h. Then, cells were centrifuged and the pellets were resuspended in PBS buffer containing lysostaphin (12.5 μg/ml; Sigma) and DNase I for 2 h. Samples were subjected to electrophoresis on SDS-PAGE 12% Criterion^TM^ TGX Stain-Free^TM^ Precast Gels (Bio-Rad). Proteins were transferred onto nitrocellulose membranes (Hybond Amersham Biosciences) and 3xFLAG fusion proteins were immunodetected by the use of anti-FLAG M2 mAbs conjugated with peroxidase (Sigma).

### Affinity Blotting of Ebh

For detection of the levels of Ebh protein overnight cultures of the different strains were diluted 1:100 in 50 ml of TSB-glu medium and grown at 37°C to a mid-log exponential phase (OD = 0.8). Surface-associated proteins were extracted as previously described (Taglialegna et al., 2016). Proteins were subjected to electrophoresis on SDS-PAGE 7.5% TGX Stain-Free^TM^ FastCast^TM^ Acrylamide Kit (Bio-Rad). For immunoblotting, proteins were transferred onto Hybond-ECL nitrocellulose membranes (Amersham Biosciences). Membranes were blocked for 2 h at room temperature with 5% skimmed milk in PBS with 0.1% Tween 20 and incubated overnight at 4°C with specific antibodies for the carboxy-terminal region of Ebh diluted 1:1,000. Bound antibodies were detected with peroxidase-conjugated goat anti-rabbit immunoglobulin G anti-bodies (Invitrogen) 1:5,000.

### Murine Model of Catheter-Associated Biofilm Formation

To examine the role of the staphylococcal TCSs in implant colonization, an *in vivo* murine model of catheter-associated biofilm formation described by Cucarella et al. (2001) was used with some modifications. *S. aureus* strains grown overnight at 37°C on TSA plates were resuspended in PBS to an optical density of OD_650 nm_ = 0.2 (10^8^ CFU/mL). Groups of 5, 5 weeks old ICR female mice (Charles River Laboratories) were anesthetized with isoflurane (B. Braun). Two 19 mm intravenous catheters (24G; B. Braun) were aseptically implanted into the subcutaneous interscapular of each mouse and inoculated with 100 μl (10^7^ CFU) of *S. aureus* strains. After 5 days, animals were opportunely euthanatized by isoflurane inhalation followed by cervical dislocation. Catheters were aseptically removed, placed in a sterile microcentrifuge tube containing 1 ml of PBS, and vortexed at high speed for 3 min. The number of bacteria was determined by plate count.

### Ethics Statement

All animal studies were reviewed and approved by the Comité de Ética para la Experimentación Animal (CEEA) of the Universidad de Navarra (approved protocol 032-17). Work was carried out at the Centro de Investigación Médica Aplicada building (ES312010000132) under the principles and guidelines described in European Directive 2010/63/EU for the protection of animals used for experimental purposes.

### Statistical Analysis

Statistical analyses were performed with the GraphPad Prism 5.01 program. The multiple comparison Tukey’s test was used in mice infection assays to find means that are significantly different from each other.

## Results

### Involvement of the *S. aureus* TCS Signaling System in the Colonization of Subcutaneous Catheters

**FIGURE 1:**
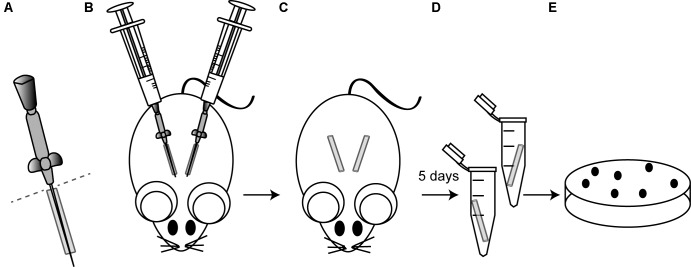
Schematic illustrating the murine model of catheter-associated biofilm formation. **(A)** ICR female mice were anesthetized by isoflurane and the skin was cleansed with alcohol prior to surgery. The bottom of 19 mm catheters (Introcan Safety 24G; B. Braun) was cut right before insertion (dotted line). **(B)** Then, two catheters were inserted into the subcutaneous interscapular space of each mice and 100 μl of the bacterial suspension containing 10^7^ CFU of the strain under study were injected through the Introcan Devices into the catheters. In all the experiments, five mice were used for each strain under study, so that a total of ten catheters were inoculated with each strain. **(C)** Introcan Devices were carefully pulled out from mice and wounds were closured with the tissue adhesive Histoacryl^®^ (B. Braun) so that catheters remained inside for 5 days. Note that although closuring the wounds, some catheters were naturally pulled out from mice during the course of experiments. **(D)** Mice were euthanized, catheters were aseptically removed, placed in a sterile microcentrifuge tube containing 1 ml of PBS and vigorously vortexed for 3 min to remove adherent bacteria. **(E)** Bacteria were enumerated by viable plate counts.

**FIGURE 2:**
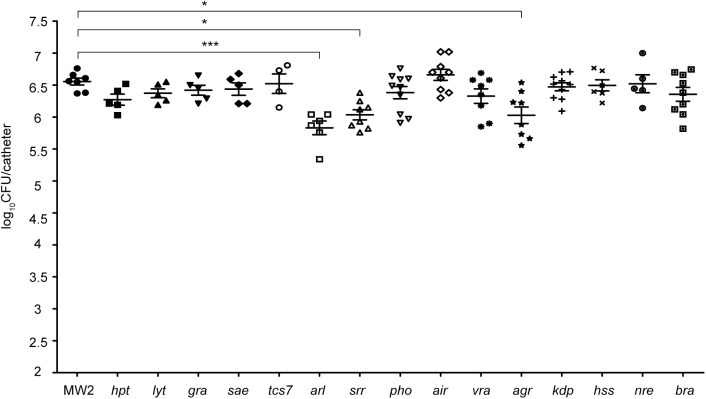
Systematic analysis of the contribution of the TCS signaling system to catheter colonization. Comparison of catheter colonization capacity of the wild type strain (MW2) and single mutants in each non-essential TCS. Bacteria were not detectable in control catheters that had been inoculated with PBS (detection limit 100 CFU/catheter). Note that although a total of ten catheters were inoculated with each strain, a variable number of catheters were recovered in each group due to natural catheter expulsion from mice during the course of the experiment. The plots display values obtained from individual catheters and the mean is represented by horizontal bars. Statistical significance was determined with one-way ANOVA followed by Tukey’s multiple comparison test comparing to the WT strain. ^∗^*P* < 0.05, ^∗∗∗^*P* < 0.001.

Since the TCS network is essential for *S. aureus* to adapt to new environments, we hypothesized that one or several TCSs might be involved in environmental sensing and activation of genes that code for biofilm-related factors needed for implant colonization. To address this question, we proceeded to systematically analyze the involvement of each TCS in the colonization of subcutaneous foreign catheters (Cucarella et al., 2001; Vergara-Irigaray et al., 2009) (**Figure 1**) by comparing the levels of catheter-associated biofilm formation of the methicillin-resistant *S. aureus* wild type MW2 strain to those of a collection of single MW2 mutants in each TCS (Villanueva et al., 2018). Because differences in growth rates could affect the capacity to colonize the catheters, we first analyzed the growth rate of each mutant under the laboratory growth conditions. The results revealed no significant differences in growth rates between the collection of mutants deficient in TCS and the wild type strain (Supplementary Figure S1). Results showed that 3 TCS single mutants, namely *arl*, *srr*, and *agr*, showed significantly decreased colonization (**Figure 2**). Interestingly, the *arl* mutant showed the greatest deficit in colonization of implanted catheters amongst all single TCS mutants. These results indicated that ArlRS, SrrAB, and Agr TCSs are concomitantly needed for biofilm formation in this DRI model, with ArlRS playing a major role in the process.

### Synthesis of the PNAG Exopolysaccharide Is Abolished in an *arl* Mutant Strain

**FIGURE 3:**
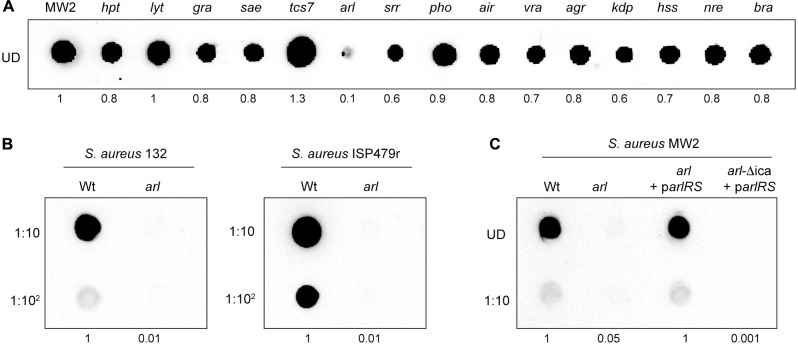
*Staphylococcus aureus arlRS* mutants do not synthesize PNAG. **(A)** Dot blot analysis of the PNAG exopolysaccharide synthesized by the wild type MW2 strain and the collection of single mutants in each non-essential TCS. **(B)** Dot blot analysis of PNAG synthesized by the wild type 132 and ISP479r strains and their corresponding *arl* mutants. **(C)** Dot blot analysis of PNAG produced by *S. aureus* MW2, a single *arl* and double *arl*-Δ*ica* mutant strains complemented with plasmid p*arlRS*, which overexpresses the *arlRS* genes under the expression of their own promoter. In all cases, samples were analyzed after 16 h of static incubation, at 37°C, in TSB (MW2 and derivatives), TSB NaCl (132) and TSBg (ISP479r). Serial dilutions (1/10) of the samples were spotted onto nitrocellulose membranes and PNAG production was detected with specific anti PIA/PNAG antibodies. UD; undiluted sample. Numbers below the image show relative dot quantification according to densitometry analysis performed with ImageJ (http://rsbweb.nih.gov/ij/).

Since the exopolysaccharide PNAG is one of the main components of the *S. aureus* biofilm matrix, we next investigated whether the deficit in catheter colonization shown by the *arl* mutant strain correlated with an altered capacity to produce PNAG. To do so, we determined PNAG production in *arl* by dot-blot, using antibodies anti-PNAG, and compared it to that of the wild type strain and the rest of single MW2 mutants in each TCS. Interestingly, the *arl* mutant strain showed a null capacity to produce PNAG. In contrast, the rest of the mutants displayed a similar, slightly higher or slightly lower PNAG production capacity than the wild type strain (**Figure 3A**). To extend the observed PNAG deficient phenotype of the *arl* mutant to other strain backgrounds, we deleted the *arlRS* genes in the genetically unrelated *S. aureus* strains 132 (methicillin-resistant) and ISP479r (methicillin-susceptible). Deficiency in PNAG production was confirmed in both *arl* mutant strains (**Figure 3B**). Moreover, complementation of the *S. aureus* MW2 *arl* mutant with the pCN51 plasmid expressing the *arl* sensor and response regulator genes under the expression of the PCad promoter (p*arlRS*) led to complete restoration of the capacity to produce PNAG (**Figure 3C**). Such restoration was completely dependent on the presence of *icaADBC* genes, since complementation of the *arl* mutant with p*arlRS* in a Δ*icaADBC* background did not result in PNAG production (**Figure 3C**). Altogether, these results showed that the ArlRS TCS is required for PNAG production in *S. aureus* and suggested a link between the incapacity of *arl* to synthesize PNAG and the deficiency shown by this mutant in the *in vivo* model of subcutaneous catheter colonization.

### Activation of PNAG Production by ArlRS Is Required for *in Vivo* Catheter Associated Biofilm Formation

**FIGURE 4:**
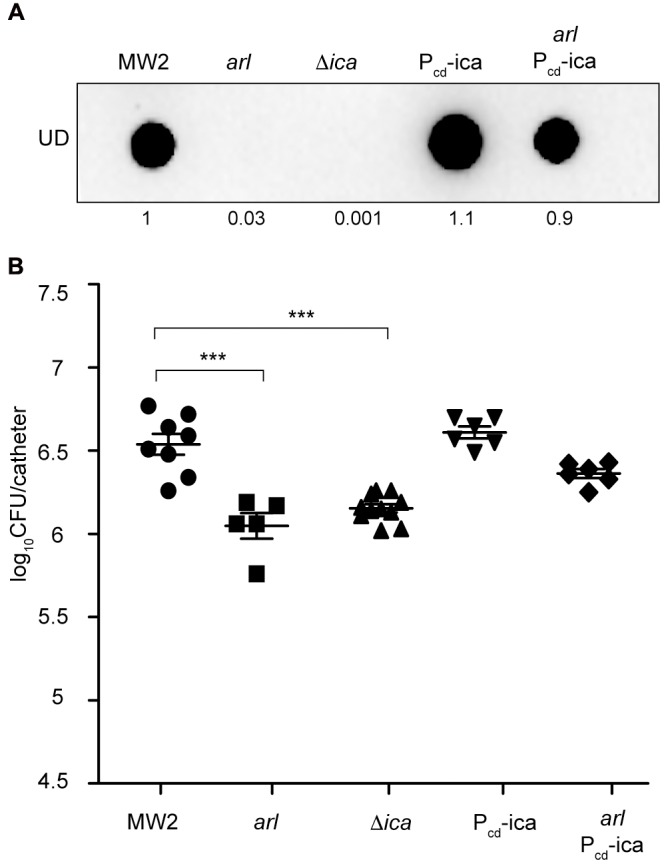
Overexpression of the *icaADBC* operon in an *arl* mutant restores catheter colonization. **(A)** Dot blot analysis of the PNAG exopolysaccharide synthesized by the wild type MW2, *arl*, Δ*ica* and the wild type and *arl* mutant that overproduce PNAG through the chromosomal expression of the *icaADBC* operon under the Pcad promoter (P_cd__ica and *arl* P_cd__ica, respectively). Samples were analyzed after 16 h of static incubation, at 37°C, in TSB media. Samples were spotted onto nitrocellulose membranes and PNAG production was detected with specific anti PIA/PNAG antibodies. Numbers below the image show relative dot quantification according to densitometry analysis performed with ImageJ (http://rsbweb.nih.gov/ij/). UD; undiluted sample. **(B)** Comparison of catheter colonization capacity of the strains shown in **(A)**. Bacteria were not detectable in control catheters that had been inoculated with PBS (detection limit 100 CFU/catheter). Note that although a total of ten catheters were inoculated with each strain, a variable number of catheters were recovered in each group due to natural catheter expulsion from mice during the course of the experiment. The plots display values obtained from individual catheters and the mean is represented by horizontal bars. Statistical significance was determined with one-way ANOVA followed by Tukey’s multiple comparison test comparing to the WT strain. ^∗∗∗^*P* < 0.001.

To investigate the possibility that catheter colonization deficiency of the *arl* mutant strain might be due to its failure to produce PNAG, we firstly analyzed catheter colonization capacity of an *arl* mutant in which the *icaADBC* operon was expressed from the chromosome, under the Pcd promoter (*arl*-P_cd__ica strain). Restoration of PNAG production in this strain (**Figure 4A**) resulted in colonization levels similar to the wild type strain (**Figure 4B**), indicating that the absence of PNAG in *arl* strain is in fact the reason for its defect in biofilm formation on subcutaneous catheters. Accordingly, a MW2 Δ*icaADBC* mutant, incapable of synthesizing PNAG (**Figure 4A**), showed a significantly reduced capacity to colonize catheters compared with the wild type strain (**Figure 4B**). Additionally, we investigated catheter colonization of *S. aureus* MW2 derivative mutants in surface proteins whose expression is known to be regulated by ArlRS and that are involved in multicellular behavior (Merino et al., 2009; Christner et al., 2010; Crosby et al., 2016). Results showed that neither protein played a role in biofilm formation on subcutaneous catheters (Supplementary Figure S2). Overall, these findings indicated that the ArlRS TCS governs the process of *S. aureus* catheter colonization through activation of the synthesis of the PNAG exopolysaccharide, which is fundamental to the formation of a biofilm on the surface of the subcutaneous implanted device.

### ArlRS Regulates *icaADBC* Expression at the Transcriptional Level

**FIGURE 5:**
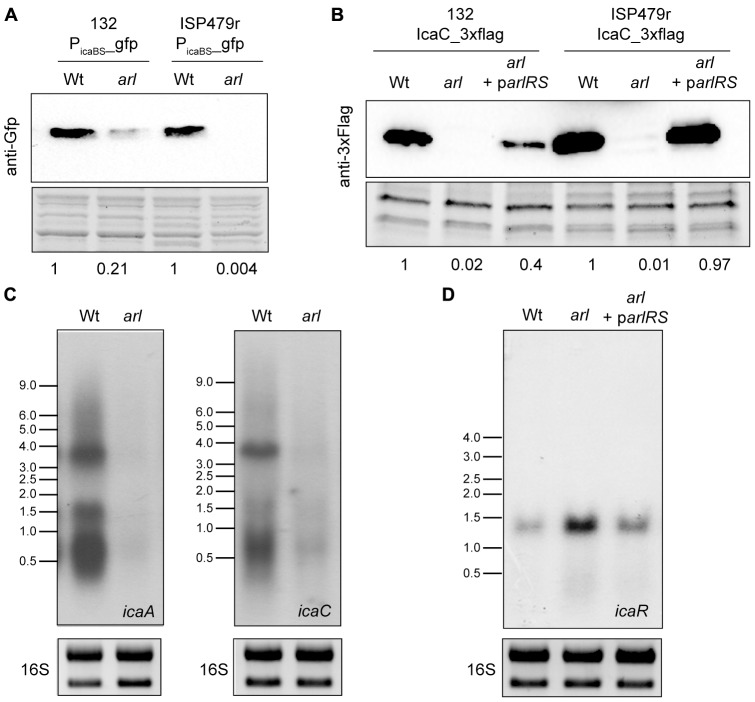
ArlRS transcriptionally activates *icaADBC* operon expression in *S. aureus*. **(A)** A representative Western blot showing GFP protein levels expressed from *S. aureus* 132 and ISP479r wild type strains and their corresponding *arl* mutants harboring plasmid P_ica(BS)_-gfp. The GFP protein was detected with commercial anti-GFP antibodies. A stain-free gel portion is shown as a loading control. Numbers below the image show relative band quantification according to densitometry analysis performed with ImageJ (http://rsbweb.nih.gov/ij/). **(B)** Representative Northern blots showing *icaA* and *icaC* mRNA of *S. aureus* 132 wild type and *arl* mutant strains grown at 37°C until exponential phase (OD_600 nm_ = 0.8). Lower panels show 16S ribosome band stained with RedSafe Nucleic Acid Staining Solution as loading control. **(C)** A representative Western blot showing IcaC protein levels of the wild type 132 and ISP479r strains, *arl* mutants and *arl* mutants complemented with a plasmid expressing the *arlRS* genes (*arl* p*arlRS*). The 3XFlag tagged IcaC protein was detected with commercial anti-3XFlag antibodies. A stain-free gel portion is shown as a loading control. Numbers below the image show relative band quantification according to densitometry analysis performed with ImageJ (http://rsbweb.nih.gov/ij/). **(D)** A representative Northern blot showing *icaR* mRNA of *S. aureus* 132 wild type strain, *arl* mutant and *arl* mutant complemented with a plasmid expressing the *arlRS* genes (*arl* p*arlRS*) grown at 37°C until exponential phase (OD_600 nm_ = 0.8). Lower panels show 16S ribosome band stained with RedSafe Nucleic Acid Staining Solution as loading control.

It has previously been shown that ArlRS regulates *S. epidermidis* biofilm formation by activating *icaADBC* operon expression, probably through repression of IcaR (Wu et al., 2012). To analyse if this is the case in *S. aureus*, a sequence upstream of the *ica* operon, including the promoter region of *icaADBC* and the IcaR binding site at the 5′UTR of *ica* genes (-147 to +30), was amplified and cloned into the pCN52 vector (Charpentier et al., 2004), which contains a promoterless *gfpmut2* gene, generating plasmid P_ica(BS)_-gfp. This plasmid was then introduced into *S. aureus* 132 and ISP479r wild type strains and their corresponding *arl* mutants, and expression of the Gfp reporter protein was determined by western-blot. Note that for this analysis, we chose to use such strains instead of *S. aureus* MW2 since as it is shown in **Figure 3**, levels of PNAG production in strains 132 and ISP479r are significantly higher than in strain MW2. Results showed that *arl* mutants expressed much lower levels of Gfp compared to wild type strains (**Figure 5A**), indicating that ArlRS activates the expression of the *icaADBC* promoter. Next, we examined, by Northern Blot, whether regulation of *ica* promoter expression by ArlRS leads to altered *icaADBC* mRNA levels, using two different riboprobes specific for *icaA* or *icaC* mRNA. Results showed a strong reduction in the levels of *icaA* and *icaC* mRNA in the *arl* mutant compared with the wild type strain (**Figure 5B**). Also, we investigated whether the decrease in the amount of *icaADBC* mRNA in the *arl* mutant correlated with lower levels of Ica proteins. To do so, we tagged the chromosomal copy of the *icaC* gene with the 3XFlag sequence in the wild type 132 and ISP479r strains, *arl* mutants and *arl* mutants complemented with a plasmid expressing the *arlRS* genes (*arl* p*arlRS*), and then, we analyzed IcaC production by Western Blot. As expected, IcaC production was abolished in *arl* mutant strains and restored to wild type levels in *arl* p*arlRS* strains (**Figure 5C**). Finally, considering that in *S. epidermidis*, Wu et al. (2012) observed an increase in *icaR* expression in an *arlRS* mutant, we wondered whether in *S. aureus*, the ArlRS TCS activates *icaADBC* operon expression through repression of IcaR. Thus, we determined the levels of *icaR m*RNA in the wild type 132 strain and compared them to the levels occurring in the *arl* mutant and the *arl* mutant complemented with a plasmid expressing the *arlRS* genes. As it is shown in **Figure 5D**, mutation of *arl* led to a significant increase in *icaR* mRNA levels that were lowered to approximately wild type levels when the *arl* mutant was complemented with the *arlRS* genes. Collectively, these findings indicated that in *S. aureus*, ArlRS regulates *icaADBC* expression at the transcriptional level, at least through downregulation of *icaR* expression, resulting in the activation of PNAG production.

### Role of MgrA in the Colonization of Subcutaneous Catheters

**FIGURE 6:**
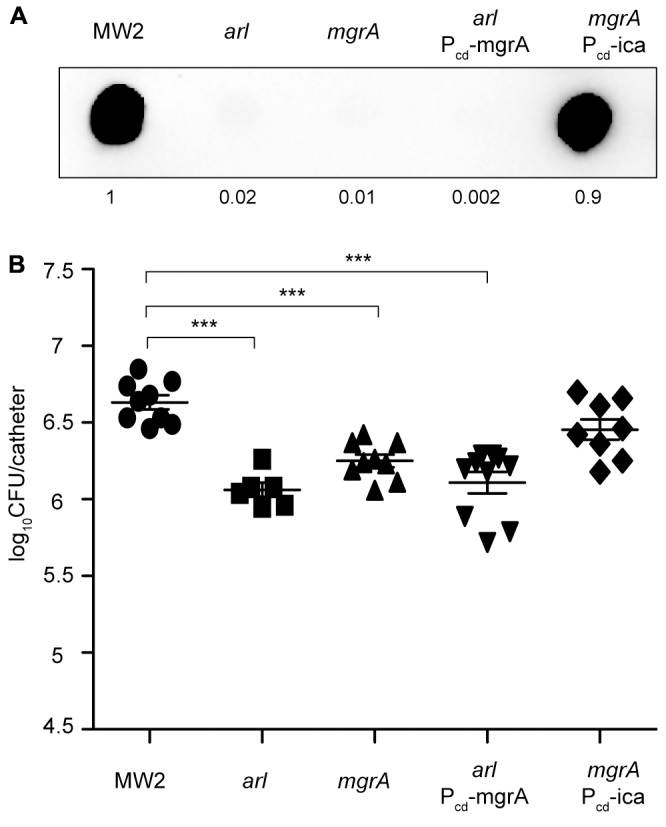
MgrA is unable to restore catheter colonization of an *arl* mutant. **(A)** Dot blot analysis of the PNAG exopolysaccharide synthesized by the wild type MW2, *arl*, *mgrA*, *arl* that overproduces MgrA through the chromosomal expression of the *mgrA* gene under the Pcad promoter (*arl* P_cd__mgrA), and *mgrA* that overproduces PNAG through the chromosomal expression of the *icaADBC* operon under the Pcad promoter (*mgrA* P_cd__ica). Samples were analyzed after 16 h of static incubation, at 37°C, in TSB media. Samples were spotted onto nitrocellulose membranes and PNAG production was detected with specific anti PIA/PNAG antibodies. Numbers below the image show relative dot quantification according to densitometry analysis performed with ImageJ (http://rsbweb.nih.gov/ij/). UD; undiluted sample. **(B)** Comparison of catheter colonization capacity of *S. aureus* strains shown in **(A)**. Bacteria were not detectable in control catheters that had been inoculated with PBS (detection limit 100 CFU/catheter). Note that although a total of 10 catheters were inoculated with each strain, a variable number of catheters were recovered in each group due to natural catheter expulsion from mice during the course of the experiment. The plots display values obtained from individual catheters and the mean is represented by horizontal bars. Statistical significance was determined with one-way ANOVA followed by Tukey’s multiple comparison test comparing to the WT strain. ^∗∗∗^*P* < 0.001.

It is known that ArlRS and the global regulator MgrA form a regulatory cascade (Supplementary Figure S3A) in which MgrA acts downstream of ArlRS to control expression of a number of genes important for virulence, including those for several large surface proteins (Crosby et al., 2016). Also, MgrA seems to regulate PNAG synthesis, since a mutant in *mgrA* has been shown to produce lower levels of PNAG than its corresponding wild type strain (Trotonda et al., 2008). Taken these results into account, we decided to investigate the contribution of MgrA to catheter colonization mediated by the ArlRS TCS. To do so, we firstly constructed a *mgrA* mutant in the MW2 strain and analyzed its capacity to synthesize PNAG by dot blot, using anti PNAG antibodies. The *mgrA* mutant lacked all capacity to produce PNAG (**Figure 6A**). We then compared catheter colonization of the *arl* mutant with that of the *mgrA* mutant and verified that both strains showed a similar deficiency in implant colonization. Importantly, catheter colonization capacity of the *mgrA* mutant was restored when the *icaADBC* operon was expressed from the chromosome, under the Pcad promoter (*mgrA*-P_cd__ica strain) (**Figure 6B**), confirming the requirement of PNAG production for implant colonization. Then, we performed epistatic experiments by overexpressing MgrA in the *arlRS* mutant (Supplementary Figure S3B) and analyzing PNAG production and colonization capacity of the resulting strain (*arl* P_cd__mgrA). Results showed that MgrA overexpression in the *arlRS* background restored neither PNAG production nor implant colonization capacity, indicating that MgrA activity cannot counteract ArlRS absence.

## Discussion

Many loss-of-function studies have been performed in several pathogenic bacteria to identify the TCSs required for virulence using *in vivo* infection models (Lasaro et al., 2014; Reboul et al., 2014; Cheng et al., 2015; Thomassin et al., 2017). However, to our knowledge, this is the first systematic study conducted to determine the role of each TCS in colonization and survival of *S. aureus* on the surface of an implanted medical device. We used a collection of single mutants in each non-essential TCS of *S. aureus* MW2 and a murine subcutaneous catheter infection model to identify TCSs controlling colonization of the implanted device. It is important to note that none of the single mutants in each TCS show a defect in growth rate when compared to the wild type strain, at least under the laboratory growth conditions tested (Supplementary Figure S1). The strongest phenotype in our screening was observed when the *arlRS* TCS was mutated, leading to a greater than 10-fold reduction in the colonization of the catheter. This result was somehow unexpected since other studies have shown that *arlRS* mutants display an increased ability to form a biofilm *in vitro*, due to the release of eDNA (Fournier and Hooper, 2000; Walker et al., 2013) and to the overexpression of SasG, a large surface protein implicated in biofilm formation (Crosby et al., 2016). Furthermore, Toledo-Arana et al. (2005) also showed that mutation of *arlRS* in *S. aureus* 15981 strain, which is naturally defective in *agr*, promoted biofilm formation in a chemically defined media (HHWm) through overexpression of the protein A surface protein. In contrast, mutation of *arlRS* in the closely related *S. epidermidis* reduces biofilm formation capacity both *in vitro* and *in viv*o (Wu et al., 2012, 2014). The molecular mechanism by which ArlRS regulates biofilm formation in *S. epidermidis* has been related with the capacity of ArlR to bind to the promoter region between *icaR* and *icaADBC* and to repress *icaR* expression, which indirectly results in an activation of *icaADBC* expression. In agreement with these results, we showed that ArlRS is involved in the regulation of *icaADBC* expression in *S. aureus*. Deletion of *arlRS* in three genetically unrelated *S. aureus* strains led to a deficiency in PNAG production in TSB media. Transcriptional fusions performed between the *icaADBC* promoter and the *gfp* reporter gene showed that the GFP levels were reduced significantly (about 30%) (**Figure 5A**) in the absence of ArlRS. In addition, *icaC* mRNA levels and consequently, IcaC protein levels, were reduced in the *arl* strain. Thus, we concluded that ArlRS regulates *icaADBC* operon expression at the level of transcription. The control that ArlRS exerts over *icaADBC* transcription might occur directly or through another regulatory protein(s). Similarly to what has been demonstrated in *S. epidermidis*, we observed that IcaR expression is upregulated in the absence of ArlRS, strongly suggesting that in *S. aureus*, regulation of *icaADBC* transcription by ArlR is mediated through upregulation of IcaR. Further studies are necessary to determine the direct interaction of ArlR with the promoter of the *icaR* gene.

It is believed that the properties provided by the biofilm lifestyle depend on the composition of the biofilm matrix, which can vary not only between strains but also within the same bacterial strain depending on the environmental conditions. *S. aureus* can form biofilms either made of the PNAG exopolysaccharide or composed by surface proteins such as fibronectin binding proteins, FnBPs, protein A, SasG or Bap (Cucarella et al., 2001; Corrigan et al., 2007; Merino et al., 2009; Vergara-Irigaray et al., 2009). Despite the mutation of *arlRS* leads to an increased expression of several surface proteins, including some involved in multicellular behavior, such as SasG, Ebh, and Spa (Fournier and Hooper, 2000; Walker et al., 2013; Crosby et al., 2016), in the present study we have shown that none of these proteins seem to play a role in the colonization of implanted catheters. On the contrary, *S. aureus* MW2 required PNAG to form a biofilm on the surface of the subcutaneous catheter. This result was again unexpected since *S. aureus* MW2 is a weak PNAG exopolysaccharide producer under laboratory conditions (Trotonda et al., 2008). This fact warns against the value of *in vitro* PNAG production analysis in order to infer PNAG relevance during infection.

In the present study, we used commercial polyurethane (PU) intravenous catheters. PU is one of the most commonly used material for catheter building due to its high excellent physical properties and good biocompatibility. Analysis of the molecular forces involved in PNAG-mediated adhesion using single-cell force spectroscopy revealed that cationic PNAG binds to anionic surfaces via multivalent electrostatic interactions (Formosa-Dague et al., 2016). During cell-to-cell interactions, it has been proposed that PNAG connects the cells together by electrostatic interaction between its positively charged groups and negatively charged molecules of wall teichoic acids on opposing cells (Gross et al., 2001; Vergara-Irigaray et al., 2008). In the case of cell-implant interaction it seems plausible that PNAG also mediates surface adhesion via electrostatic interactions. On the other hand, adhesion to biological surfaces is often mediated by hydrophobic interactions that are usually the strongest of all long-range non-covalent forces (Briandet et al., 2001; Cerca et al., 2005). It is known that hydrophobic materials favor bacterial adherence more than hydrophilic ones and consequently modification of the bacterial surface hydrophobicity can also affect the capacity to colonize the catheter surface (Pavithra and Doble, 2008). We have not addressed whether surface hydrophobicity is modified in the absence of ArlRS and future studies to clarify this possibility are needed. On the other hand, though bacteria can encounter the naked implant surface, we also need to consider that immediately following the implantation of the catheter, a layer of host proteins rapidly adsorbs on its surface, altering the properties of the polymer which may affect bacterial adhesion and biofilm formation. In our experimental model, catheters were infected immediately after implantation without allowing the catheter to be coated with plasma proteins. This model mimics those infection processes that occur during catheter implantation but we cannot exclude that colonization of a catheter surface coated by plasma may involve additional factors.

ArlR activates expression of one of the promoters of the transcriptional regulator MgrA, also called Rat or NorR (Fournier et al., 2000; Ingavale et al., 2003; Crosby et al., 2016). This global regulator modulates the expression of 5–10% of the *S. aureus* genome, and a large part of the ArlRS regulon depends on the expression of MgrA (Luong et al., 2003; Gupta et al., 2015; Crosby et al., 2016). Based on results shown by Trotonda et al. (2008) that indicate that although *S. aureus* mutants in *mgrA* have increased *in vitro* biofilm formation capacity, they produce lower levels of the PNAG exopolysaccharide, we hypothesized that MgrA might be related with the *arlRS* mutant deficiency in catheter colonization. Our results confirmed that an *mgrA* mutant has a decreased PNAG production capacity and also showed that in our catheter infection model, the *mgrA* mutant presents a colonization deficit similar to that of the *arlRS* mutant. Importantly, complementation of both *arlRS* and *mgrA* mutants with the *icaADBC* operon restored implant colonization capacity. However, epistatic experiments revealed that overexpression of MgrA in the *arl* mutant restored neither PNAG production nor implant colonization capacity. These results confirmed the impact that the PNAG exopolysaccharide has for catheter colonization and also indicated that as regards PNAG expression, MgrA activity cannot compensate for ArlRS absence. One plausible explanation for this might be that both regulators regulate *icaADBC* expression at a different level. According to Wu et al. (2012) and to our results, ArlRS regulates *icaADBC* expression at the transcriptional level through the *icaR* repressor. Instead, MgrA seems to regulate *icaADBC* expression at the posttranscriptional level (Trotonda et al., 2008). These last observations may explain why the *ica* genes have never been identified in studies carried out to define the MgrA regulon (Luong et al., 2003; Crosby et al., 2016).

Our results also showed that deletion of Agr and SrrAB TCSs cause a slight reduction in the capacity to colonize subcutaneous catheters. Agr controls biofilm architecture and cell dispersion through the regulation of phenol soluble modulins (PSMs) in a *ica*-independent manner (Otto, 2012). PSMs can aggregate and form amyloid fibrils that contribute to stability of the biofilm (Schwartz et al., 2012, 2014). Because the levels of the PNAG exopolysaccharide produced *in vitro* were similar between the wild type and *agr* mutant strains, it is likely that PNAG is not involved in the decreased colonization capacity of the *agr* mutant *in vivo*. On the other hand, SrrAB has been shown to induce biofilm formation under anaerobic conditions through activation of *icaADBC* gene transcription (Ulrich et al., 2007; Wu et al., 2015) and release of extracellular DNA (Mashruwala et al., 2017). Since implanted catheters are thought to comprise anaerobic microenvironments, it might be possible that the *srrA* mutant has a catheter colonization deficiency because of a defect linked to oxygen sensing.

In summary, we have shown that ArlRS plays a significant role in *S. aureus* implant colonization by affecting the expression of the *icaADBC* operon by directly modifying *icaR* transcription. Although different studies using different genetic strains coincide in showing that ArlRS plays an important role in biofilm mediated infections, it is also noticeable the strong strain and growth media dependency in the observed phenotypes. Thus, additional studies are necessary to determine the precise mechanisms of regulation and the signals to which this TCS responds.

## Author Contributions

SB, CS, IL, and JV designed the work and revised the manuscript. SB, CG, and JV completed all the experiments. SB and CG performed the statistically analysis and made the figures. JV, SB, IL, and CS wrote the manuscript.

## Conflict of Interest Statement

The authors declare that the research was conducted in the absence of any commercial or financial relationships that could be construed as a potential conflict of interest.

## References

[B1] Alanidis C. Dejong P. J. (1990). Ligation-independent cloning of PCR products (LIC-PCR). *Nucleic Acids Res.* 18 6069–6074. 10.1093/nar/18.20.6069 2235490 PMC332407

[B2] Arnaud M. Chastanet A. Débarbouillé M. (2004). New vector for efficient allelic replacement in naturally nontransformable, low-GC-content, gram-positive bacteria. *Appl. Environ. Microbiol.* 70 6887–6891. 10.1128/AEM.70.11.6887-6891.2004 15528558 PMC525206

[B3] Briandet R. Herry J. M. Bellon-Fontaine M. N. (2001). Determination of the van der Waals, electron donor and electron acceptor surface tension components of static Gram-positive microbial biofilms. *Colloids Surf. B Biointerfaces* 21 299–310. 10.1016/S0927-7765(00)00213-7 11397632

[B4] Cerca N. Pier G. B. Vilanova M. Oliveira R. Azeredo J. (2005). Quantitative analysis of adhesion and biofilm formation on hydrophilic and hydrophobic surfaces of clinical isolates of *Staphylococcus epidermidis*. *Res. Microbiol.* 156 506–514. 10.1016/j.resmic.2005.01.007 15862449 PMC1356821

[B5] Charpentier E. Anton A. I. Barry P. Alfonso B. Fang Y. Novick R. P. (2004). Novel cassette-based shuttle vector system for gram-positive bacteria. *Appl. Environ. Microbiol.* 70 6076–6085. 10.1128/AEM.70.10.6076-6085.2004 15466553 PMC522135

[B6] Cheng A. T. Ottemann K. M. Yildiz F. H. (2015). *Vibrio cholerae* response regulator VxrB controls colonization and regulates the type VI secretion system. *PLoS Pathog.* 11:e1004933. 10.1371/journal.ppat.1004933 26000450 PMC4441509

[B7] Cheung A. L. Bayer A. S. Zhang G. Gresham H. Xiong Y.-Q. (2004). Regulation of virulence determinants in vitro and in vivo in *Staphylococcus aureus*. *FEMS Immunol. Med. Microbiol.* 40 1–9. 14734180 10.1016/S0928-8244(03)00309-2

[B8] Christner M. Franke G. C. Schommer N. N. Wendt U. Wegert K. Pehle P. (2010). The giant extracellular matrix-binding protein of *Staphylococcus epidermidis* mediates biofilm accumulation and attachment to fibronectin. *Mol. Microbiol.* 75 187–207. 10.1111/j.1365-2958.2009.06981.x 19943904

[B9] Corrigan R. M. Rigby D. Handley P. Foster T. J. (2007). The role of *Staphylococcus aureus* surface protein SasG in adherence and biofilm formation. *Microbiology* 153 2435–2446. 10.1099/mic.0.2007/006676-0 17660408

[B10] Crosby H. A. Schlievert P. M. Merriman J. A. King J. M. Salgado-Pabón W. Horswill A. R. (2016). The *Staphylococcus aureus* global regulator MgrA modulates clumping and virulence by controlling surface protein expression. *PLoS Pathog.* 12:e1005604. 10.1371/journal.ppat.1005604 27144398 PMC4856396

[B11] Cucarella C. Solano C. Valle J. Amorena B. Lasa I. Penadés J. R. (2001). Bap, a *Staphylococcus aureus* surface protein involved in biofilm formation. *J. Bacteriol.* 183 2888–2896. 10.1128/JB.183.9.2888-2896.2001 11292810 PMC99507

[B12] Ellis M. W. Schlett C. D. Millar E. V. Crawford K. B. Cui T. Lanier J. B. (2014). Prevalence of nasal colonization and strain concordance in patients with community-associated *Staphylococcus aureus* skin and soft-tissue infections. *Infect. Control Hosp. Epidemiol.* 35 1251–1256. 10.1086/678060 25203178 PMC5824647

[B13] Formosa-Dague C. Speziale P. Foster T. J. Geoghegan J. A. Dufrêne Y. F. (2016). Zinc-dependent mechanical properties of *Staphylococcus aureus* biofilm-forming surface protein SasG. *Proc. Natl. Acad. Sci. U.S.A.* 113 410–415. 10.1073/pnas.1519265113 26715750 PMC4720321

[B14] Foster T. J. Geoghegan J. A. Ganesh V. K. Höök M. (2014). Adhesion, invasion and evasion: the many functions of the surface proteins of *Staphylococcus aureus*. *Nat. Rev. Microbiol.* 12 49–62. 10.1038/nrmicro3161 24336184 PMC5708296

[B15] Fournier B. Aras R. Hooper D. C. (2000). Expression of the multidrug resistance transporter NorA from *Staphylococcus aureus* is modified by a two-component regulatory system. *J. Bacteriol.* 182 664–671. 10.1128/JB.182.3.664-671.2000 10633099 PMC94328

[B16] Fournier B. Hooper D. C. (2000). A new two-component regulatory system involved in adhesion, autolysis, and extracellular proteolytic activity of *Staphylococcus aureus*. *J. Bacteriol.* 182 3955–3964. 10.1128/JB.182.14.3955-3964.2000 10869073 PMC94580

[B17] Gross M. Cramton S. E. Götz F. Peschel A. (2001). Key role of teichoic acid net charge in *Staphylococcus aureus* colonization of artificial surfaces. *Infect. Immun.* 69 3423–3426. 10.1128/IAI.69.5.3423-3426.2001 11292767 PMC98303

[B18] Gupta R. K. Luong T. T. Lee C. Y. (2015). RNAIII of the *Staphylococcus aureus* agr system activates global regulator MgrA by stabilizing mRNA. *Proc. Natl. Acad. Sci. U.S.A.* 112 14036–14041. 10.1073/pnas.1509251112 26504242 PMC4653210

[B19] Haag A. F. Bagnoli F. (2016). “The role of two-component signal transduction systems in *Staphylococcus aureus* virulence regulation,” in *Current Topics in Microbiology and Immunology*, eds Bagnoli F. Rappuoli R. Grandi G. (Berlin: Springer), 1–54. 10.1007/82_2015_5019 26728068

[B20] Hogan S. Stevens N. T. Humphreys H. O’Gara J. P. O’Neill E. (2015). Current and future approaches to the prevention and treatment of staphylococcal medical device-related infections. *Curr. Pharm. Des.* 21 100–113. 25189861 10.2174/1381612820666140905123900

[B21] Ingavale S. S. van Wamel W. Cheung A. L. (2003). Characterization of RAT, an autolysis regulator in *Staphylococcus aureus*. *Mol. Microbiol.* 48 1451–1466. 10.1046/j.1365-2958.2003.03503.x 12791130

[B22] Kluytmans J. van Belkum A. Verbrugh H. (1997). Nasal carriage of *Staphylococcus aureus*: epidemiology, underlying mechanisms, and associated risks. *Clin. Microbiol. Rev.* 10 505–520. 9227864 10.1128/cmr.10.3.505 PMC172932

[B23] Kuroda M. Ohta T. Uchiyama I. Baba T. Yuzawa H. Kobayashi I. (2001). Whole genome sequencing of meticillin-resistant *Staphylococcus aureus*. *Lancet* 357 1225–1240. 11418146 10.1016/s0140-6736(00)04403-2

[B24] Lasa I. Toledo-Arana A. Dobin A. Villanueva M. de los Mozos I. R. Vergara-Irigaray M. (2011). Genome-wide antisense transcription drives mRNA processing in bacteria. *Proc. Natl. Acad. Sci. U.S.A* 108 20172–20177. 10.1073/pnas.1113521108 22123973 PMC3250193

[B25] Lasaro M. Liu Z. Bishar R. Kelly K. Chattopadhyay S. Paul S. (2014). *Escherichia coli* isolate for studying colonization of the mouse intestine and its application to two-component signaling knockouts. *J. Bacteriol.* 196 1723–1732. 10.1128/JB.01296-13 24563035 PMC3993324

[B26] Lowy F. D. (1998). *Staphylococcus aureus* infections. *N. Engl. J. Med.* 339 520–532. 10.1056/NEJM199808203390806 9709046

[B27] Luong T. T. Newell S. W. Lee C. Y. (2003). Mgr, a novel global regulator in *Staphylococcus aureus*. *J. Bacteriol.* 185 3703–3710. 12813062 10.1128/JB.185.13.3703-3710.2003 PMC161569

[B28] Maira-Litrán T. Kropec A. Abeygunawardana C. Joyce J. Mark G. Goldmann D. A. (2002). Immunochemical properties of the staphylococcal poly-N-acetylglucosamine surface polysaccharide. *Infect. Immun.* 70 4433–4440. 12117954 10.1128/IAI.70.8.4433-4440.2002 PMC128161

[B29] Mashruwala A. A. Gries C. M. Scherr T. D. Kielian T. Boyd J. M. (2017). SaeRS is responsive to cellular respiratory status and regulates fermentative biofilm formation in *Staphylococcus aureus*. *Infect. Immun.* 85:e00157–17. 10.1128/IAI.00157-17 28507069 PMC5520442

[B30] Merino N. Toledo-Arana A. Vergara-Irigaray M. Valle J. Solano C. Calvo E. (2009). Protein A-mediated multicellular behavior in *Staphylococcus aureus*. *J. Bacteriol.* 191 832–843. 10.1128/JB.01222-08 19047354 PMC2632097

[B31] Novick R. P. (2003). Autoinduction and signal transduction in the regulation of staphylococcal virulence. *Mol. Microbiol.* 48 1429–1449. 10.1046/j.1365-2958.2003.03526.x 12791129

[B32] O’Gara J. P. (2007). *ica* and beyond: biofilm mechanisms and regulation in *Staphylococcus epidermidis* and *Staphylococcus aureus*. *FEMS Microbiol. Lett.* 270 179–188. 10.1111/j.1574-6968.2007.00688.x 17419768

[B33] O’Neill E. Pozzi C. Houston P. Humphreys H. Robinson D. A. Loughman A. (2008). A novel *Staphylococcus aureus* biofilm phenotype mediated by the fibronectin-binding proteins, FnBPA and FnBPB. *J. Bacteriol.* 190 3835–3850. 10.1128/JB.00167-08 18375547 PMC2395027

[B34] Otto M. (2012). Molecular basis of *Staphylococcus epidermidis* infections. *Semin. Immunopathol.* 34 201–214. 10.1007/s00281-011-0296-2 22095240 PMC3272124

[B35] Pavithra D. Doble M. (2008). Biofilm formation, bacterial adhesion and host response on polymeric implants—issues and prevention. *Biomed. Mater.* 3 34003–34014. 10.1088/1748-6041/3/3/034003 18689922

[B36] Reboul A. Lemaître N. Titecat M. Merchez M. Deloison G. Ricard I. (2014). *Yersinia pestis* requires the 2-component regulatory system OmpR-EnvZ to resist innate immunity during the early and late stages of plague. *J. Infect. Dis.* 210 1367–1375. 10.1093/infdis/jiu274 24813471

[B37] Rice K. C. Mann E. E. Endres J. L. Weiss E. C. Cassat J. E. Smeltzer M. S. (2007). The cidA murein hydrolase regulator contributes to DNA release and biofilm development in *Staphylococcus aureus*. *Proc. Natl. Acad. Sci. U.S.A.* 104 8113–8118. 10.1073/pnas.0610226104 17452642 PMC1876580

[B38] Ruiz de Los Mozos I. Vergara-Irigaray M. Segura V. Villanueva M. Bitarte N. Saramago M. (2013). Base pairing interaction between 5′- and 3′-UTRs controls icaR mRNA translation in *Staphylococcus aureus*. *PLoS Genet.* 9:e1004001. 10.1371/journal.pgen.1004001 24367275 PMC3868564

[B39] Schenk S. Laddaga R. A. (1992). Improved method for electroporation of *Staphylococcus aureus*. *FEMS Microbiol. Lett.* 73 133–138. 1521761 10.1016/0378-1097(92)90596-g

[B40] Schwartz K. Sekedat M. D. Syed A. K. O’Hara B. Payne D. E. Lamb A. (2014). The AgrD N-Terminal leader peptide of *Staphylococcus aureus* has cytolytic and amyloidogenic properties. *Infect. Immun.* 82 3837–3844. 10.1128/IAI.02111-14 24980969 PMC4187843

[B41] Schwartz K. Syed A. K. Stephenson R. E. Rickard A. H. Boles B. R. (2012). Functional amyloids composed of phenol soluble modulins stabilize *Staphylococcus aureus* biofilms. *PLoS Pathog.* 8:e1002744. 10.1371/journal.ppat.1002744 22685403 PMC3369951

[B42] Stock A. M. Robinson V. L. Goudreau P. N. (2000). Two-component signal transduction. *Annu. Rev. Biochem.* 69 183–215. 10.1146/annurev.biochem.69.1.183 10966457

[B43] Taglialegna A. Navarro S. Ventura S. Garnett J. A. Matthews S. Penadés J. R. (2016). Staphylococcal Bap proteins build amyloid scaffold biofilm matrices in response to environmental signals. *PLoS Pathog.* 12:e1005711. 10.1371/journal.ppat.1005711 27327765 PMC4915627

[B44] Thomassin J.-L. Leclerc J.-M. Giannakopoulou N. Zhu L. Salmon K. Portt A. (2017). Systematic analysis of two-component systems in *Citrobacter rodentium* reveals positive and negative roles in virulence. *Infect. Immun.* 85:e00654–16. 10.1128/IAI.00654-16 27872242 PMC5278178

[B45] Toledo-Arana A. Merino N. Vergara-Irigaray M. Débarbouillé M. Penadés J. R. Lasa I. (2005). *Staphylococcus aureus* develops an alternative, ica-independent biofilm in the absence of the arlRS two-component system. *J. Bacteriol.* 187 5318–5329. 10.1128/JB.187.15.5318-5329.2005 16030226 PMC1196035

[B46] Trotonda M. P. Tamber S. Memmi G. Cheung A. L. (2008). MgrA represses biofilm formation in *Staphylococcus aureus*. *Infect. Immun.* 76 5645–5654. 10.1128/IAI.00735-08 18852246 PMC2583562

[B47] Ulrich M. Bastian M. Cramton S. E. Ziegler K. Pragman A. A. Bragonzi A. (2007). The staphylococcal respiratory response regulator SrrAB induces *ica* gene transcription and polysaccharide intercellular adhesin expression, protecting *Staphylococcus aureus* from neutrophil killing under anaerobic growth conditions. *Mol. Microbiol.* 65 1276–1287. 10.1111/j.1365-2958.2007.05863.x 17697253

[B48] Valle J. Toledo-Arana A. Berasain C. Ghigo J.-M. Amorena B. Penadés J. R. (2003). SarA and not sigmaB is essential for biofilm development by *Staphylococcus aureus*. *Mol. Microbiol.* 48 1075–1087. 12753197 10.1046/j.1365-2958.2003.03493.x

[B49] Vergara-Irigaray M. Maira-Litran T. Pier G. B. Penadés J. R. Lasa I. (2008). Wall teichoic acids are dispensable for anchoring the PNAG exopolysaccharide to the *Staphylococcus aureus* cell surface. *Microbiology* 154 865–877. 10.1099/mic.0.2007/013292-0 18310032 PMC2292800

[B50] Vergara-Irigaray M. Valle J. Merino N. Latasa C. García B. Ruiz de Los Mozos I. (2009). Relevant role of fibronectin-binding proteins in *Staphylococcus aureus* biofilm-associated foreign-body infections. *Infect. Immun.* 77 3978–3991. 10.1128/IAI.00616-09 19581398 PMC2738049

[B51] Villanueva M. García B. Valle J. de los Mozos I. R. Solano C. Rapun B. (2018). Sensory deprivation in *Staphylococcus aureus*. *Nat. Commun.* 9:523. 10.1038/s41467-018-02949-y 29410457 PMC5802764

[B52] Walker J. N. Crosby H. A. Spaulding A. R. Salgado-Pabón W. Malone C. L. Rosenthal C. B. (2013). The *Staphylococcus aureus* ArlRS two-component system is a novel regulator of agglutination and pathogenesis. *PLoS Pathog.* 9:e1003819. 10.1371/journal.ppat.1003819 24367264 PMC3868527

[B53] Wu Y. Liu J. Jiang J. Hu J. Xu T. Wang J. (2014). Role of the two-component regulatory system *arlRS* in ica operon and *aap* positive but non-biofilm-forming *Staphylococcus epidermidis* isolates from hospitalized patients. *Microb. Pathog.* 76 89–98. 10.1016/j.micpath.2014.09.013 25263000

[B54] Wu Y. Wang J. Xu T. Liu J. Yu W. Lou Q. (2012). The two-component signal transduction system ArlRS regulates *Staphylococcus epidermidis* biofilm formation in an *ica*-dependent manner. *PLoS One* 7:e40041. 10.1371/journal.pone.0040041 22848368 PMC3407220

[B55] Wu Y. Wu Y. Zhu T. Han H. Liu H. Xu T. (2015). *Staphylococcus epidermidis* SrrAB regulates bacterial growth and biofilm formation differently under oxic and microaerobic conditions. *J. Bacteriol.* 197 459–476. 10.1128/JB.02231-14 25404696 PMC4285975

