# Supplementary material for: A Systematic Evaluation of the Two-Component Systems Network Reveals That ArlRS Is a Key Regulator of Catheter Colonization by Staphylococcus aureus
Source: Front Microbiol. 2018 Mar 7;9:342. doi: 10.3389/fmicb.2018.00342 (PMC5845881; doi:10.3389/fmicb.2018.00342)
Supplement: Supplementary file 1 [file Table_1.PDF]

Table S1. Strains and plasmids used in this study

Strains	Description	MIC	Reference
Staphylococcus aureus			
MW2	Community-acquired strain of MRSA, which was isolated in 1998 in North Dakota, USA.	3566	(Baba *et al.*, 2008)
132	MRSA clinical strain	29	(Vergara-Irigaray *et al.*, 2009)
Isp479r	ISP479c with *rsbU* gene restored	1680	(Toledo-Arana *et al.*, 2005)
MW2 *hpt*	MW2 Δ*hptRS*	4032	(Villanueva *et al.*, 2018)
MW2 *lyt*	MW2 Δ*lytSR*	2964	(Villanueva *et al.*, 2018)
MW2 *gra*	MW2 Δ*graRS*	11	(Villanueva *et al.*, 2018)
MW2 *sae*	MW2 Δ*saeRS*	2965	(Villanueva *et al.*, 2018)
MW2 *tcs7*	MW2 Δ*MW1208-MW1209*	4033	(Villanueva *et al.*, 2018)
MW2 *arl*	MW2 Δ*arlRS*	4034	(Villanueva *et al.*, 2018)
MW2 *srr*	MW2 Δ*srrAB*	2966	(Villanueva *et al.*, 2018)
MW2 *pho*	MW2 Δ*phoPR*	4035	(Villanueva *et al.*, 2018)
MW2 *air*	MW2 Δ*airSR*	3670	(Villanueva *et al.*, 2018)
MW2 vra	MW2 Δ*vraSR*	4036	(Villanueva *et al.*, 2018)
MW2 *agr*	MW2 Δ*agrBDCA*	4037	(Villanueva *et al.*, 2018)
MW2 *kdp*	MW2 Δ*kdpDE*	4038	(Villanueva *et al.*, 2018)
MW2 *hss*	MW2 Δ*hssRS*	2979	(Villanueva *et al.*, 2018)
MW2 *nre*	MW2 Δ*nreBC*	2967	(Villanueva *et al.*, 2018)
MW2 *bra*	MW2 Δ*braRS*	4039	(Villanueva *et al.*, 2018)
MW2 *arl*+p*arlRS*	MW2 Δ*arl* carrying pCN51::*arlRS* plasmid	4539	(Villanueva *et al.*, 2018)
MW2 *arl* Δica+p*arlRS*	MW2 Δ*arl* with a deletion of the ica operon carrying pCn51::*arlRS* plasmid	6642	This study
132 *arl*	132 with a deletion of the *arlRS* genes	6050	This study
132 *arl* p*arlRS*	132 Δ*arl* carrying pCN51::*arlRS* plasmid	6568	This study
Isp479r *arl*	ISP479r with a deletion of the *arlRS* genes	1921	(Toledo-Arana *et al.*, 2005)
Isp479r *arl* p*arlRS*	Isp479r Δarl carrying pCN51::*arlRS* plasmid	6569	This study
132 $P_{ica(BS)}$-*gfp*	132 carrying pCN52::$P_{ica(BS)}$-gfp plasmid	6011	This study
132 *arl* $P_{ica(BS)}$-*gfp*	132 Δ*arl* carrying pCN52::$P_{ica(BS)}$-gfp plasmid	6639	This study
Isp479r $P_{ica(BS)}$-*gfp*	Isp479r carrying pCN52::$P_{ica(BS)}$-gfp plasmid	6032	This study
Isp479r *arl* $P_{ica(BS)}$-*gfp*	Isp479r Δ*arl* carrying pCN52::$P_{ica(BS)}$-gfp plasmid	6640	This study
132 IcaC_3xflag	132 carrying a *3xFLAG* epitope in IcaC	2731	This study
132 *arl* IcaC_3xflag	132 Δ*arl* carrying a 3xFLAG epitope in IcaC	6547	This study
132 *arl* IcaC_3xflag+p*arlRS*	132 Δ*arl* carrying a 3xFLAG epitope in IcaC and pCN51::*arlRS* plasmid	6548	This study
Isp479r IcaC_3xflag	Isp479r carrying a 3xFLAG epitope in IcaC	2641	This study
Isp479r *arl* IcaC_3xflag	Isp479r Δ*arl* carrying a 3xFLAG epitope in IcaC	6549	This study
Isp479r *arl* IcaC_3xflag+p*arlRS*	Isp479r Δ*arl* carrying a 3xFLAG epitope in IcaC and pCN51::*arlRS* plasmid	6550	This study
MW2 Δ*ica*	MW2 with a deletion of the *ica* operon	6367	This study
MW2 P_{Cd}-*ica*	MW2 expressing the *ica* operon from the chromosome under de cadmium inducible promoter	6370	This study
MW2 *arl* P_{Cd}-*ica*	MW2 Δ*arl* expressing the *ica* operon from the chromosome under de cadmium inducible promoter	6546	This study
MW2 P_{mgra}-*gfp*	MW2 carrying pCN52::P_{mgrA}-gfp plasmid	5990	This study
MW2 *arl* P_{mgra}-*gfp*	MW2 Δ*arl* carrying pCN52::P_{mgrA}-gfp plasmid	5991	This study
MW2 *mgrA*	MW2 with a deletion of the *mgrA* gene	6643	This study
MW2 *arl* P_{Cd}-*mgrA*	MW2 Δ*arl* expressing the *mgrA* gene from the chromosome under de cadmium inducible promoter	6644	This study
MW2 *mgrA* P_{Cd}-*ica*	MW2 Δ*mgrA* expressing the *ica* operon from the chromosome under de cadmium inducible promoter	6645	This study
MW2 *ebh*	MW2 with a deletion from -196 to +774	6646	This study

MW2 *spa*	MW2 with a deletion of the *spa* gene	5003	This study
MW2 *sasG*	MW2 with a deletion of the *sasG* gene	6647	This study

Plasmids	Description	Reference
pMAD	E. coli-S. aureus shuttle vector with a thermosensitive origin of replication for gram-positive bacteria	(Arnaud *et al.*, 2004)
pCN51	*E. coli* - *S. aureus* shuttle vector to express genes under the control of the Pcd cadmium-inducible promoter. Em^R	(Charpentier *et al.*, 2004)
p*arlRS*	pCN51 plasmid expressing *arlRS* genes	(Villanueva *et al.*, 2018)
pCN52	*E. coli* - *S. aureus* shuttle vector with promoterless gfpmut2. Em^R	(Charpentier *et al.*, 2004)
$P_{ica(BS)}$-gfp	pCN52 plasmid expressing gfpmut2 under the *ica* promoter	This study
P_{mgrA}-gfp	pCN52 plasmid expressing gfpmut2 under the *mgrA* promoter	This study
pMAD::*ica*AD	pMAD plasmid containing the allele for deletion of 2684 bp of the *ica* operon	(Toledo-Arana *et al.*, 2005)
pMAD::*icaC*_3xflag	pMAD_lic plasmid containing the allele for insertion of the 3xflag epitope at the C-terminal of IcaC	This study
pMAD_lic	pMAD modified plasmid for inserting DNA fragments using a ligase independent cloning	This study
pMAD_lic::P_{Cd}_*ica*	pMAD_lic plasmid containing the allele for insertion of the cadmium-inducible promoter before the *icaADBC* mRNA	This study
pMAD_lic::*mgrA*-AD	pMAD_lic plasmid containing the allele for deletion of the *mgrA* gene	This study
pMAD_lic::P_{Cd}_*mgrA*	pMAD_lic plasmid containing the allele for insertion of the cadmium-inducible promoter before the *mgrA* mRNA	This study
pMAD_lic::*ebh*-AD	pMAD_lic plasmid containing the allele for *ebh* deletion from -196 to +774	This study
pMAD::*spa*-AD	pMAD plasmid containing the allele for deletion of the *spa* gene	(Merino *et al.*, 2009)
pMAD::*sasG*-AD	pMAD plasmid containing the allele for deletion of the *sasG* gene	(Vergara-Irigaray *et al.*, 2009)

Arnaud, M., Chastanet, A., and Débarbouillé, M. (2004) New vector for efficient allelic replacement in naturally nontransformable, low-GC-content, gram-positive bacteria. *Appl Environ Microbiol* **70**: 6887–6891.

Baba, T., Bae, T., Schneewind, O., Takeuchi, F., and Hiramatsu, K. (2008) Genome sequence of *Staphylococcus aureus* strain Newman and comparative analysis of staphylococcal genomes: polymorphism and evolution of two major pathogenicity islands. *J Bacteriol* **190**: 300–310.

Charpentier, E., Anton, A.I., Barry, P., Alfonso, B., Fang, Y., and Novick, R.P. (2004) Novel Cassette-Based Shuttle Vector System for Gram-Positive Bacteria. *Appl Environ Microbiol* **70**: 6076–6085.

Merino, N., Toledo-Arana, A., Vergara-Irigaray, M., Valle, J., Solano, C., Calvo, E., *et al.* (2009) Protein A-mediated multicellular behavior in *Staphylococcus aureus. J Bacteriol* **191**: 832–843.

Toledo-Arana, A., Merino, N., Vergara-Irigaray, M., Débarbouillé, M., Penadés, J.R., and Lasa, I. (2005) *Staphylococcus aureus* develops an alternative, ica-independent biofilm in the absence of the arlRS two-component system. *J Bacteriol* **187**: 5318–5329.

Vergara-Irigaray, M., Valle, J., Merino, N., Latasa, C., García, B., Ruiz de Los Mozos, I., *et al.* (2009) Relevant role of fibronectin-binding proteins in *Staphylococcus aureus* biofilm-associated foreign-body infections. *Infect Immun* **77**: 3978–3991.

Villanueva, M., García, B., Valle, J., de los Mozos, I. R., Solano, C., Rapun, B., et al. (2018). Sensory deprivation in *Staphylococcus aureus*. Nature Communications, in press
