# Supplementary material for: A Systematic Evaluation of the Two-Component Systems Network Reveals That ArlRS Is a Key Regulator of Catheter Colonization by Staphylococcus aureus
Source: Front Microbiol. 2018 Mar 7;9:342. doi: 10.3389/fmicb.2018.00342 (PMC5845881; doi:10.3389/fmicb.2018.00342)
Supplement: Supplementary file 2 [file Table_2.PDF]

Table S2. Oligonucleotides used in this study

Oligonucleotide	Sequence 5´→3´
**pMAD_lic**	
pMAD_lic (EcoRI)	GAATTCTGACGACGACAAGAGGGCCC
pMAD_lic (BamHI)	GGATCCTGAGGAGAAGCCCGGGGCCC
**Transcriptional expression of *ica* and *mgra* promoters**	
AU59	ATGCCTGCAGGTCGACTTTTTATAACCCCCTACTGAAAATTAATCACACT
AU76	ACGAATTCGAGCTCGGTACCTTTCTTTACCTACCTTTCGTTAGTTAGGTTG
PmgrA-fw	ATGCCTGCAGGTCGACGTCCCCTTTTAAAGCAATGGC
PmgrA-rv	ACGAATTCGAGCTCGAATAAGAATATCCATAATTAACGGATTTTTGGGTAGT
**IcaC 3xflag epitope insertion**	
CFLAG-A	GTCGACGCAAATGGAGACTATTG
CFLAG-B	GCGGCCGCTTACTATTTATCGTCGTCATCTTTGTAGTCGATATCATGATCTTTATAATCACCGTCATGG TCTTTGTAGTCATAAGCATTAATGTTCAATTTA
CFLAG-C	GCGGCCGCTTATTAAGCTATGTTAAAAAC
CFLAG-D	CCATGGGCACAAGAGAAGAATTAC
CFLAG-E	ATGAAAAAGATTAGACTTGAACT
CFLAG-F	CTATTTATCGTCGTCATCTTTGTAGTCG
**Cadmium promoter insertion before *icaADBC* operon**	
Pica_LIC-A	GACGACGACAAGAGTCTTATTCTTTTCAGGGGAAC
PCd-ica-B	TGAATAAGTGCGTTTCTTTACCTACCTTTCGT
Ica-Cd-fw	TAGGTAAAGAAACGCACTTATTCAAGTGTATTTTT
Ica-Cd-rv	AAAAAATTGCAATGCAGGTTCAGACATTG
PCd-ica-C	TCTGAACCTGCATTGCAATTTTTTAACTTTTTGC
Pica_LIC-D	GAGGAGAAGCCCGGTCTTGATCAACGATAGTATCTG
Cd-fw	CTAGCTTTATATTCTTTAGGTG
AU7	GGATCCGCACCAAGTTTTGGATCA
**Cadmium promoter insertion before *mgrA* gene**	
PmgrA_LIC-A	GACGACGACAAGAGTCGTCCCCTTTTAAAGCAATG
PCd-mgrA-B	AATAAGTGCGTAAAGTTCTCCTCCAGACATAC
mgrA-Cd-fw	GGAGAACTTTACGCACTTATTCAAGTGTATTTTT
mgrA-Cd-rv	AATAAGTGCGTAAAGTTCTCCTCCAGACATAC
PCd-mgrA-C	GAACCTGCAATGTCTGATCAACATAATTTAAAAG
PmgrA_LIC-D	GAGGAGAAGCCCGGTTTATTTTTCCTTTGTTTCATCAAATG
Cd-fw	CTAGCTTTATATTCTTTAGGTG
Mgra-F	GATGAAAAAGATGAAGCGGT
**Deletion of *mgra, ebh, sasG* and *spa* genes**	
mgrA LIC-A	GGAGGAGAACTTTATTAACTTTTGTCATGACAATTAAAGTAATG
mgra-B	ATGACAAAAGTTAATAAAGTTCTCCTCCAGACATACTAT
mgra-C	GGAGGAGAACTTTATTAACTTTTGTCATGACAATTAAAGTAATG
mgrA LIC-D	GAGGAGAAGCCCGGTGGCACTAGAACGTCAAATTGAC
mgrA-E	GACATGCAACTAGTAATTCCA
mgrA-F	GATGAAAAAGATGAAGCGGT
Ebh-LIC-A	GACGACGACAAGAGTTAGGTTATTTATTCTTTGGTTTAGGAC
Ebh-B	ATTCTGTTTGAGCTCCAGAATATAATAACACAAAATATATT
Ebh-C	TTATATTCTGGAGCTCAAACAGAATTCAAACGCG
ebh-LIC-D	GAGGAGAAGCCCGGTTCCATATTCCCACATTTGTTGA
ebh-E	GAGTTCTTATTGTCGGAGAATG
ebh-F	TGGCCACTTCTATTTCTTATTTT
**Riboprobes**	
AU52	TAATACGACTCACTATAGGGTATCCACGTAAATGCAATTTCC
AU53	TGGAAGTTCAGATAATACAGC
AU54	TAATACGACTCACTATAGGGGAATTCACGCAATATCAT
AU55	TCACGATACCGTGCTACAC
RP T7 sense icaR	TAATACGACTCACTATAGGGTACTTTCTTCCACTGCTCCA
icaR +1 (BamHI)	GGATCCGAAATATTTGTAATTGCAACTTA
