# Supplementary material for: A Systematic Evaluation of the Two-Component Systems Network Reveals That ArlRS Is a Key Regulator of Catheter Colonization by Staphylococcus aureus
Source: Front Microbiol. 2018 Mar 7;9:342. doi: 10.3389/fmicb.2018.00342 (PMC5845881; doi:10.3389/fmicb.2018.00342)
Supplement: Supplementary file 3 [file Image_1.PDF]

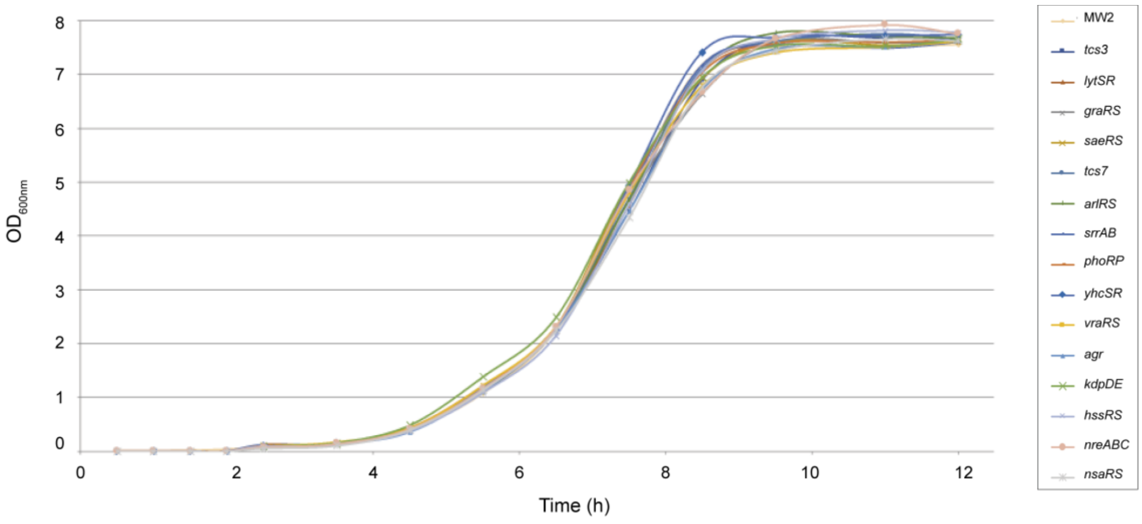

Figure S1. Growth kinetics of MW2 wild-type and their respective mutants in TCSs.

Growth curves of S. aureus MW2 and the mutants in TCSs grown in TSB-gluc medium at 37ºC. OD600nm of the culture was measured every 30 minutes during 12 h.
