# Supplementary material for: A Systematic Evaluation of the Two-Component Systems Network Reveals That ArlRS Is a Key Regulator of Catheter Colonization by Staphylococcus aureus
Source: Front Microbiol. 2018 Mar 7;9:342. doi: 10.3389/fmicb.2018.00342 (PMC5845881; doi:10.3389/fmicb.2018.00342)
Supplement: Supplementary file 4 [file Image_2.PDF]

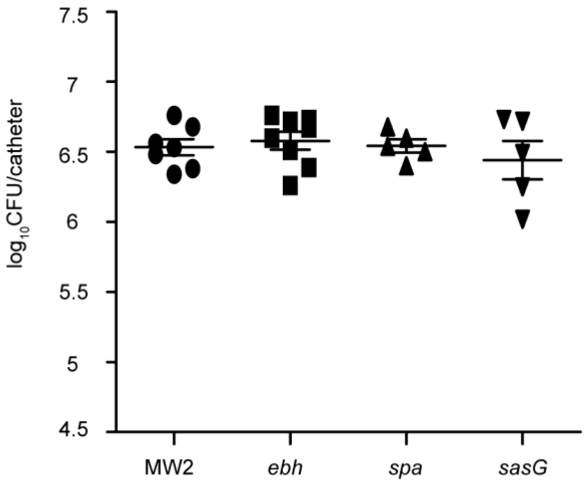

Figure S2. Comparison of catheter colonization capacity of mutants in surface proteins whose expression is regulated by ArlRS. Comparison of catheter colonization capacity of the wild type strain (MW2) and single mutants in *ebh*, *spa* and *sasG*. Bacteria were not detectable in control catheters that had been inoculated with PBS (detection limit 100 CFU/catheter). Note that although a total of ten catheters were contaminated with each strain, a variable number of catheters were recovered in each group due to natural catheter expulsion from mice during the course of the experiment. The plots display values obtained from individual catheters and the mean is represented by horizontal bars. Statistical significance was determined with one-way ANOVA followed by Tukey's multiple comparison test comparing to the WT strain. In all cases, differences were not statistically significant.
