# Supplementary material for: A Systematic Evaluation of the Two-Component Systems Network Reveals That ArlRS Is a Key Regulator of Catheter Colonization by Staphylococcus aureus
Source: Front Microbiol. 2018 Mar 7;9:342. doi: 10.3389/fmicb.2018.00342 (PMC5845881; doi:10.3389/fmicb.2018.00342)
Supplement: Supplementary file 5 [file Image_3.pdf]

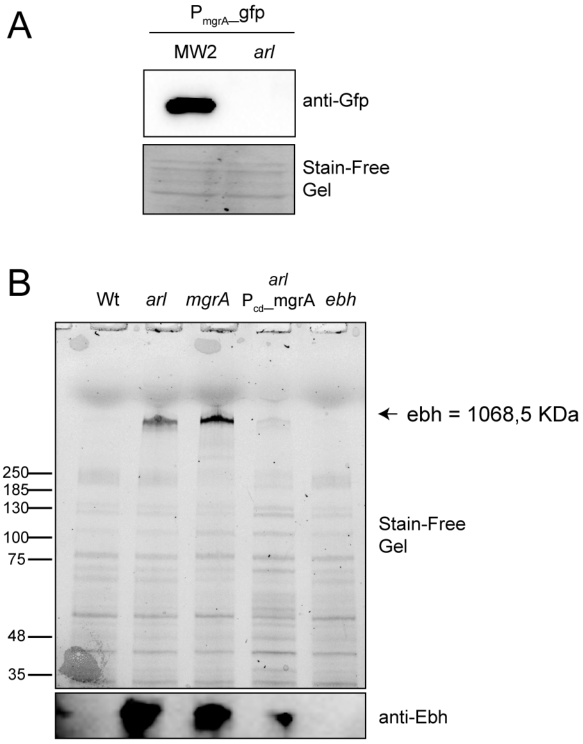

Figure S3. ArlRS controls MgrA expression in *S. aureus* MW2. (A) The P2 promoter of *mgrA* is activated by ArlRS. A representative Western blot showing GFP protein levels expressed from *S. aureus* MW2 wild type and *arl* mutant strains harboring plasmid P_{mgrA}_gfp, which contains the P2 promoter region of *mgrA* fused to a promoterless *gfpmut2* gene. The GFP protein was detected with commercial anti-GFP antibodies. A stain-free gel portion is shown as a loading control. **(B)** Confirmation of the complementation of MgrA expression in *arl* P_{cd}_mgrA strain. It is known that ArlRS represses the expression of the cell wall-associated protein Ebh through MgrA activation. Thus, a deletion of either *arl* or *mgrA* should lead to Ebh overexpression whilst complementation of an *arl* mutant with *mgrA* should result in a repression of Ebh production. Ebh protein levels expressed from *S. aureus* strains MW2 wild type, *arl*, *mgrA* and *arl* that overproduces MgrA through the chromosomal expression of the *mgrA* gene under the Pcad promoter (*arl* P_{cd}_mgrA) were analyzed by SDS-PAGE and Western

blot, using anti-Ebh antibodies. A mutant in *ebh* was included as a control of no Ebh production. Cell wall extracts were taken at exponential phase ($OD_{600\ nm}$ = 0.8) from strains grown in TSBg at 37ºC.
